# Assessing dairy cow welfare during the grazing and housing periods on spring-calving, pasture-based dairy farms

**DOI:** 10.1093/jas/skab093

**Published:** 2021-03-24

**Authors:** Robin E Crossley, Eddie A M Bokkers, Natasha Browne, Katie Sugrue, Emer Kennedy, Imke J M de Boer, Muireann Conneely

**Affiliations:** 1 Teagasc, Animal and Grassland Research and Innovation Centre, Moorepark, Fermoy, Co. Cork P61 C996, Ireland; 2 Animal Production Systems Group, Department of Animal Sciences, Wageningen University & Research, 6700 AH, Wageningen, the Netherlands; 3 School of Veterinary Medicine and Science, University of Nottingham, Loughborough, United Kingdom

**Keywords:** animal-based, avoidance behavior, body condition, cattle, health, lameness

## Abstract

The different periods characterizing spring-calving, pasture-based dairy systems common in Ireland have seldom been the focus of large-scale dairy cow welfare research. Thus, the aim of this study was to devise and conduct an animal-based welfare assessment during both the grazing and housing periods on spring-calving, pasture-based dairy farms, to identify areas for improvement and establish benchmarks for indicators of good welfare. Assessment of seven animal-based welfare indicators was conducted during two visits (one each at grazing and housing) to 82 commercial dairy farms in southern Ireland. Herd-level descriptive statistics were performed for all welfare indicators at each visit, and differences between visits were analyzed using paired *t*-tests and Wilcoxon signed-rank tests. A mean of 9% and 10% clinically lame cows (mobility scores 2 and 3) were observed at housing and grazing, respectively. Recommended body condition scores (**BCS**) were not met for a mean of 13% of cows at grazing and 23% at housing, with more over-conditioned cows present at housing than grazing (*P* < 0.001). Ocular discharge was uncommon in both periods. Prevalence of moderate and severe nasal discharge combined was lower during housing (5%) than grazing (7%). In both periods, similar mean levels of tail injury were observed: 2% to 3% of cows with tail lacerations, 9% with broken tails, and 8% (measured at housing only) with docked tails. Integument alterations involved primarily hair loss and were most prevalent on the hindquarters (26%) during grazing and on the head–neck–back (66%) and the hindquarter (32%) regions during housing. Cows displayed an avoidance distance of >1 m (indicative of a fearful response) from an approaching human in an average of 82% of grazing cows and 42% to 75% of housed cows, dependent on test location. Opportunities to improve welfare in this system were identified in the areas of tail injury prevention, nasal health, and the management of indoor housing and feeding. The performance of the top 20% of farms for each welfare indicator was used to establish benchmarks of: 0% to 5% clinical lameness, 0% to 12% of cows outside recommended BCS, 0% to 27% ocular discharge, 2% to 16% nasal discharge, 0% tail lacerations and docked tails, 0% to 3% tail breaks, 0% to 14% integument alterations, and 4% to 74% for avoidance distance of >1 m. These represent attainable targets for spring-calving pasture-based farms to promote good dairy cow welfare.

## Introduction

Maintaining good animal welfare is a vital component of a sustainable dairy production system. Not only to sustain continued support from the public but also because producers have an ethical responsibility to ensure that the animals under their care are free from unnecessary suffering and able to experience a life worth living. Optimal welfare requires that animals have all the necessities to maintain good biological health and function, are able to perform important and highly motivated natural behaviors, and are able to experience an overall positive affective state (Fraser, 2008). What most of the on-farm welfare assessment protocols have in common is their use of animal-based indicators of welfare, chosen because they directly reflect the experience of a cow within her environment as opposed to the resources provided (Whay et al., 2003; Webster et al., 2004). However, the majority of existing dairy cow welfare assessment protocols have been designed to evaluate animals when housed and thus do not take into account the grazing period in pasture-based dairy production systems.

Whereas there are many advantages of access to pasture on dairy cow welfare, including the reduced prevalence of lameness (Olmos et al., 2009) and mastitis (Washburn et al., 2002) and the ability to perform natural grazing behaviors, there are also challenges. Cows at pasture have a greater risk of internal parasites (Mee, 2012) and are exposed to inclement weather, such as rain, wind, or heat, rarely with access to shelter (Van laer et al., 2014). When cows are transferred indoors for the winter housing period, they must contend with additional challenges, such as a change in diet from grazed pasture to silage (O′Driscoll et al., 2008), introduction to concrete or slatted flooring, which may negatively affect claw health (Cook et al., 2004), reduced air quality (Casey et al., 2006), and reduced feeding and lying space allowance, which may lead to increased agonistic behavior (Kondo et al., 1989).

To date, there has been little large-scale research into the welfare of dairy cows encompassing both phases of pasture-based systems, which include a period of housing. Most of the previous studies of these systems have been small in scale, such as Olmos et al. (2009), which focused on groups of cows on a single Irish farm managed either at pasture or in continuous housing. Burow et al. (2013) evaluated 41 Danish herds, yet with a minimum access to pasture of only 5 h/d for ≥ 120 d/yr. Wagner et al. (2018), who studied a combination of 32 organic and conventional herds in Germany, categorized pasture access time between 0 and ≥ 12 h/d, and all farms were provided additional silage year-round. In contrast, cows on a typical Irish pasture-based dairy farm are at pasture 24 h/d for an average of 229 d/yr (Teagasc, 2019).

The paucity of dairy cow welfare data available for Irish pasture-based systems is particularly noteworthy considering that the Irish dairy sector has undergone substantial growth in recent years resulting from the elimination of European Union milk production quotas in 2015. Since this time in Ireland, farm sizes have increased by 7% to an average of 59 ha, herd sizes have increased by 13% to an average of 80 cows/farm, and the total number of dairy cows in the country has increased by 21% to 1.4 million animals in 2019 (Hennessy and Moran, 2015; Donnellan et al., 2020). Such expansion throughout the sector poses a potential risk to dairy cow welfare if management practices and infrastructure are not adapted to the continually expanding herd and farm sizes.

In order to optimize cow welfare and ensure a sustainable dairy sector, it is vital to understand the impact of current on-farm management practices. Therefore, the objective of this study was to devise and conduct an animal-based welfare assessment during both the grazing and housing periods on a large cross-section of Irish, spring-calving, pasture-based dairy farms. Through descriptive analysis of different welfare measures, this study further aims to identify areas for improvement and establish benchmarks for various animal-based indicators of welfare.

## Materials and Methods

All experimental procedures received ethical approval from the Teagasc Animal Ethics Committee (TAEC 197-2018) and were conducted in accordance with the Cruelty to Animals Act (Ireland 1876, as amended by European Communities regulations 2002 and 2005) and the European Community Directive 86/609/EC. All statistical procedures were carried out using SAS 9.4 (SAS Institute Inc., Cary, NC, USA).

### Farm selection criteria

This study includes data from 82 commercial dairy farms in the Republic of Ireland, visited twice between April 2019 and February 2020. Farms were selected from the Irish Cattle Breeding Federation (**ICBF**), the national information database for the dairy and beef farming sector. All farms operated a conventional (non-organic), pasture-based (>200 d/yr grazing grass), primarily spring-calving system (≤7% cows calving out-of-season from July to November 2019), consisting of primarily cross-bred dairy breeds (majority Holstein, Friesian, and Jersey cross) and purebred Holstein cows. The target herd size was 30 to 250 cows, which represented 95% of all farms meeting the above criteria. All farms were selected from within the primary dairy-producing counties of Ireland: Cork, Tipperary, Limerick, Kerry, Kilkenny, Waterford, and Wexford, which account for 69% of all dairy cows in the country (Central Statistics Office, 2020). For feasibility, all farms were also within a 2-h driving distance from the Teagasc Moorepark research center, located in Fermoy, Cork, Ireland.

### Farm recruitment

An initial target of 100 farms was determined as the maximum number of farms that could reasonably be visited within the time frame, and with the resources available, while still providing as representative a sample as possible. Using the SURVEYSELECT procedure, 500 farms were randomly selected from an initial list of 3,388 farms meeting the selection criteria. These farms were sent a letter outlining the study and inviting them to contact the research team if interested in participating. A total of 121 farms replied (24% response rate). Nine of these were deemed unable to facilitate the study procedures, and a further 10 declined to participate. The first 100 eligible farms were selected for enrollment in the study. In the final weeks of the study, seven initially enrolled farms were no longer available to participate. The two remaining farms from the first round of selection were contacted. A further 18 farms were randomly selected from the pool of remaining farms using the RANDBETWEEN procedure, and they were invited by phone to participate (56% response rate) in order to source the seven required farms.

In total, 103 farms were visited during the grazing season (**VISIT1**; April to September 2019). A second visit was planned for all farms during the housing period (**VISIT2**; October 2019 to February 2020) once their herd had transitioned to full-time housing (i.e., cows housed 24 hr/d). However, some farmers decided to withdraw from the study prior to VISIT2; therefore, only 87 farms were revisited during this period. The mean interval between VISIT1 and VISIT2 was 168 ± 53.6 d (range 65 to 262 d).

Of the 103 total visited farms, two farms were excluded from the study as they were found, upon arrival, to milk cows only once per day. A third farm was excluded because it operated a robotic milking system that prevented us from collecting data in a manner consistent with the other study farms. A final four farms were excluded because too many cows calved out of season to meet the spring-calving criteria. For the purposes of this study, only those farms from which we were able to collect data for both the summer grazing period and winter housing period were included, resulting in a total of 82 farms.

### Farm visit procedure

At VISIT1, one farm was assessed each day, either during or immediately following morning milking. Visits began with animal scoring, followed by a behavioral test of the cows and a management survey completed with the farmer. At VISIT2, the team was able to assess two farms per day (one each in the morning and afternoon) because of a shorter visit duration and more flexibility in start time, as many farms had finished milking or had altered milking schedules during dry-off. The same animal scoring procedure was conducted as in VISIT1, followed by a modified behavioral test and a management survey specific to VISIT2.

Animal scoring consisted of mobility (**MS**), body condition (**BCS**), and health scoring. To facilitate animal scoring, all cows passed sequentially through a handling area consisting of a race with a cow-restraining gate from which each animal was released individually. Mobility scoring was performed by one observer as each cow exited the handling area. Simultaneously, a second observer performed the BCS and health scoring on a subset of cows within the handling area as they waited to be released. The number of cows scored was proportionate to herd size according to the sample size selection table utilized by Welfare Quality (2009). Depending on the farm facilities, the cows either proceeded directly out to their destination paddock following scoring or were retained in a holding yard until later released by the farmer.

### Farm visit team

Teams of three to four trained observers were present at each farm visit. All team members were similarly dressed, in dark-colored clothing (brown/dark blue) and adhered to a biosecurity protocol at the start and end of each farm visit, ensuring fresh, disinfected equipment. Any members of the team tasked with conducting MS attended a training course from the UK Agriculture and Horticulture Development Board (**AHDB**) and were successfully accredited by the UK Register of Mobility Scorers. Furthermore, tests of observer reliability were carried out for all team members tasked with either MS or BCS on farms. Scores of agreement between observers (inter-observer reliability) were calculated as weighted kappa coefficients, with a target mean score of ≥0.7. Inter-observer reliability agreement was tested twice for each of MS and BCS; once at the start of VISIT1 (mean MS agreement score = 0.73, range: 0.62 to 0.84; mean BCS agreement score = 0.74, range: 0.66 to 0.82), and once at the start of VISIT2 (mean MS agreement score = 0.85, range: 0.80 to 0.91; mean BCS agreement score = 0.81, range: 0.73 to 0.86). An additional test of BCS inter-observer reliability was conducted part-way through VISIT1, when a new scorer was introduced (mean BCS agreement score = 0.77, range: 0.67 to 0.84). Intra-observer reliability (agreement within scorers) was tested at the start of VISIT2 for MS (mean agreement score = 0.77, range: 0.71 to 0.81) and BCS (mean agreement score = 0.87, range: 0.81 to 0.93).

### Data collection

#### Animal scoring—VISIT1 and VISIT2

All lactating cows were assessed on each farm at VISIT1. At VISIT2, both lactating and dry cows that were part of the 2019 lactating group were assessed. At both visits, all eligible cows were scored for mobility, while only a subset of cows proportionate to herd size (Welfare Quality, 2009) were scored for body condition, health scores, and behavior.

Mobility scoring utilized the AHDB 4-point scale (Agriculture and Horticulture Development Board, 2020a): 0, walking well with no visible gait imperfections; 1, an imperfect gait requiring further monitoring, but where no intervention was yet required; 2, an identifiable problem with the cow’s gait in one or more limbs where intervention was required as soon as possible; and 3, severely impaired mobility requiring immediate intervention. Those scored MS0 were considered not-lame, those scored MS1 were considered to have imperfect mobility, and those scoring MS2 (moderately lame) or MS3 (severely lame) were together considered clinically lame. All clinically lame cows received appropriate treatment by a trained hoof-care professional (Farm Relief Services Network, Derryvale Roscrea Co. Tipperary, Ireland) within 11 d of their assessment as part of a concurrent study (Browne et al., unpublished).

Body condition scoring was conducted using a 5-point scale between 1 and 5 with increments of 0.25 (Agriculture and Horticulture Development Board, 2020b). Score 1 indicates an emaciated cow requiring immediate attention and score 5 indicates an extremely over-conditioned animal requiring managed weight loss to maintain good health.

Health scoring consisted of assessing an individual cow’s eyes, nose, tail, and integument for signs of poor health or injury. The same subset of cows was scored for health as BCS at each visit. Both ocular and nasal discharges were scored on 4-point scales adapted from the University of Wisconsin-Madison calf-health scoring system (https://fyi.extension.wisc.edu/heifermgmt/files/2015/02/calf_health_scoring_chart.pdf). Eyes received an ocular score (**OS**) from 0, “normal,” to 3, “heavy” ocular discharge. A nasal score (**NS**) was assessed between 0, “normal serous discharge,” and 3, “copious bilateral mucopurulent discharge.” The tail was manually assessed for signs of injury, including circumferential lacerations, breakage, and docking (both short, removal of the tail approximately at the level of the rear udder attachment, and long, removal of the tail just above the distal end to prevent long hair growth).

Integument was assessed for hair loss and the presence of lesions and/or swelling according to a scoring key adapted from Welfare Quality (2009). One full side of each cow was visually divided into five zones, each assessed for the presence of either single, multiple, or no areas of integument alteration. The alterations were further assigned a score corresponding to the greatest level of severity: 0) no missing, thinning or broken hair, and no swelling; 1) bald area with none or very mild swelling; 2) medium swelling (1 to 2.5 cm) and/or a lesion (scab or open wound) present; and 3) major swelling (>2.5 cm), with or without the presence of a lesion. The side of the animal scored was dictated by the design and direction of the handling area through which the cows were scored and, therefore, varied between farms.

#### Avoidance behavior test—VISIT1

During VISIT1, an avoidance behavior test was conducted with the cows while at pasture. The test, adapted from Rousing and Waiblinger (2004), measured the avoidance response to an approaching human. Subsequent to animal scoring, a trained observer followed the cows to the paddock. The observer selected a standing cow > 2 m ahead of their location and, while slowly approaching her head (approximately 1 step/s), attempted to touch each cow according to a standardized procedure. The observer recorded the point at which each cow first showed signs of retreat (backed away or turned head away to either side) at five different levels: 1) >2 m from the observer, 2) within 1 to 2 m from the observer, 3) within 1 m from the observer but before extending hand, 4) accepting of extended hand but not touch, and 5) accepting of touch. The cows’ responses to the human approach were further categorized into Fearful (levels 1 and 2), Intermediate (level 3), or Non-fearful (levels 4 and 5).

#### Avoidance behavior test—VISIT2

The avoidance test at VISIT2 followed the same procedure as in VISIT1 with some modifications accounting for the indoor environment. The proportion of cows scored was still determined by the Welfare Quality (2009) sample size criteria; however, if the cows were housed in multiple groups, the total number of cows to score was divided proportionately between each group. Because all cows were not always engaged in the same activities within the shed at each visit, it was necessary to score cows both at the feed-face (**FF**) and within the pen. The avoidance test was performed from outside the pen for cows at the FF and from inside the pen for cows within the pen areas. The location of the tested animal (FF or pen) was recorded to account for any observed differences between the two test areas. Due to the more confined space of the shed compared with the paddock, the tag number was recorded for each scored cow to prevent duplicate scoring. No cows were encouraged to rise if lying. If there was an insufficient number of cows available to reach the target number, a minimum of 30 cows on each farm were scored. If this was not possible, the test was deemed incomplete and excluded from analysis.

### Management survey and ICBF data

During VISIT1, a survey was completed with each farmer regarding general farm characteristics, management practices, animal health, and farm infrastructure. A second follow-up survey was completed during VISIT2 to identify any changes that occurred between visits and to obtain the details of cow management at dry-off and during the housing period. Additionally, herd health and production details were collected from the ICBF database for each participating farm. Acquired data consisted of general herd information, milk recording, and fertility data from January 2019 to June 2020.

### Data management

All data were recorded on either paper worksheets or, when possible, using a handheld device (Psion Teklogix, Workabout Pro). The integument scores of one farm at VISIT1 were excluded from analysis due to recording errors. Fourteen farms at VISIT1 were excluded from the analysis of avoidance behavior; 10 of which could not be completed due to safety concerns (e.g., the presence of a bull and inclement weather) and 4 due to recording errors. At VISIT2, seven farms were excluded from behavior analysis that could not be completed because either less than the minimum of 30 cows were available for scoring or the research team was short-staffed.

Benchmarks were established for each welfare indicator by ranking the outcomes of all farms from best to worst performance for each visit. The rankings were divided into quintiles, with the top and bottom 20% of farms serving as benchmarks identifying the best and worst performing farms for each indicator. While the 20% upper and lower thresholds may be arbitrary, we believe that they provide good distinction between the highest and lowest performing farms and facilitate meaningful comparisons. Mobility rank was determined by the combined percentage of MS2 and MS3 scored cows. The BCS ranking was determined based on the percentage of cows meeting the target levels of 2.75 to 3.25 during grazing and 3 to 3.5 during housing (Butler, 2016). OS and NS ranks were determined by the combined percentage of cows scoring OS1 to OS3 or NS1 to NS3. For tail injuries, the prevalence of lacerations, breaks, and docks was ranked individually. For integument, the mean total number of alterations observed in all zones was combined to create a single ranking. The avoidance levels representing the “Fearful” response (levels 1 and 2) were combined and ranked by the total percentage.

### Statistical analysis

Data were summarized on a herd level, the percentage of each score was calculated for individual farms, and the mean percentage of each score was calculated across all study farms using the FREQ and SUMMARY procedures. The mean, standard error, minimum and maximum values, median, and 95% confidence intervals are reported for all animal scores at each of VISIT1 and VISIT2.

To determine the differences between scores at VISIT1 and VISIT2, all scores were first checked for normality using the UNIVARIATE procedure. A paired *t*-test (TTEST procedure) was used for all variables with normally distributed mean differences, whereas the Wilcoxon signed-rank test (within the UNIVARIATE procedure) was used for all non-normally distributed variables. A Bonferroni correction for multiple comparisons was calculated for indicators with multiple score levels. This involved multiplying the *P*-value resulting from each individual comparison by the total number of tests conducted for each indicator; any *P*-values > 1 were rounded to 1. For all tests, significance was declared at *P* < 0.05.

Additionally, data were examined for trends in the timing of individual farm visits within each of the VISIT1 and VISIT2 collection periods. Each visit timeframe was divided into two equal periods based on the timing of the visit. For VISIT1, period A included farms visited from April to June (*n* = 36) and period B included farms visited from July to September (*n* = 46). For VISIT2, period A included farms visited from October to mid-December (*n* = 45) and period B included farms visited from mid-December to February (*n* = 37). The GLM procedure was used with score level as the response variable and time period as the explanatory variable. The Bonferroni correction was applied for multiple comparisons, and time effects are only reported where significant.

## Results and Discussion

Consensus in the literature is that animal-based indicators provide the best measure of welfare because they most accurately reflect the animals’ experience; thus, there are a wide range of different measures used in current welfare assessment protocols (Andreasen et al., 2014; Zuliani et al., 2018). However, one challenge with assessing welfare in a system where little previous data have been collected on the associated indicators is determining acceptable welfare targets. Much of the available data for pasture-based systems throughout the world are specific to the characteristics of that particular system (Zuliani et al., 2018). The aim of the current study was to examine a representative group of farms reflecting the Irish national average of 80 cows/herd (Donnellan et al., 2020), producing 5,438 liters of milk/cow at an overall stocking rate among top performing herds of 2.48 cows/ha and a grazing season length of 229 d/yr (Teagasc, 2019). However, the resulting herd characteristics of the study farms are more representative of Irish dairy herds with above-average size and performance (Table 1). The voluntary participation required in the current study, and the fact that smaller, lower performing herds may be less likely to be members of the ICBF database from which farms were selected, may account for why the study farms represent above-average herds. However, given that expansion within the dairy sector is likely to continue, the greater average size and performance of study farms reflect this upward trend and suggest that the identified results will be applicable in the future as well. In fact, 68% of farms surveyed indicated that they had expanded in either herd size, farm size, or infrastructure over the past 5 years, and 50% indicated that they have plans to or are considering expansion in the next 5 years.

**Table 1.** Herd characteristics for spring-calving, pasture-based farms in southern Ireland, visited for welfare assessment during the grazing (VISIT1) and housing (VISIT2) periods of the 2019–2020 season

Herd characteristic	No. of farms^1^	Mean	SD	Min	Max	Median
Herd size	82	125	49.1	38	253	120
Farm size, Ha	81	45	19.2	14	101	40
Milk 305 d yield, kg/cow	72	6,706	752.2	4,013	8,251	6,769
Fat 305 d yield, kg/cow	72	280	31.4	187	367	281
Protein 305 d yield, kg/cow	72	242	26.5	157	291	241
Stocking rate						
Grazing platform, cows/ha	82	2.97	0.94	1.2	5.7	3
Cubicles, cubicles/cow	81	1	0.18	0.6	1.8	1
Loose-housing, m^2^/cow	13	5.6	2.97	2.2	11.5	5.3
Grazing Season length (full time, 24 h/d)	81	240	27.6	170	312	236
Days on grass at VISIT1	82	126	44.4	36	213	129
Days housed at VISIT2	82	39	25.1	0^2^	99	59

^1^Total of 82 study farms. Data for some characteristics were unavailable for some farms.

^2^One farm was visited on its first full day of housing.

Welfare assessment protocols often rely on expert opinion to aggregate welfare outcomes and establish target levels for measured indicators. However, aggregation can sometimes allow the effect of one indicator to overshadow the effects of others in the combined score (de Vries et al., 2013). This potential problem can be avoided by considering the welfare impact of each indicator on its own. Another increasingly common method is to establish individual benchmarks collected from on-farm animal-based measures of welfare (Main et al., 2014; Zuliani et al., 2018; Kaurivi et al., 2020). This method alone does not address existing systemic welfare issues or set limits for acceptable levels; however, it does provide many advantages for improving welfare on farms. Benchmarking thresholds encourage continuous welfare improvement within a particular system (Main et al., 2014), they are critical in identifying points at which action is required (Zuliani et al., 2018), and they promote the sharing of best practices among farmers to achieve enhanced welfare (Von Keyserlingk et al., 2012). In the current study, the range displayed by the top performing 20% farms for each indicator (Table 2) was identified as current achievable levels that may serve as a benchmark for other farms within the system. Conversely, if farms exhibit comparable levels to the bottom-ranked farms, this indicates that they are underperforming their peers operating within the same system and reveals an opportunity for achievable improvement.

**Table 2.** Range in the outcome percentage of scored cows for each welfare indicator among the top and bottom performing 20% of study farms during grazing (VISIT1) and when housed (VISIT2)

	Grazing				Housed			
	Top 20%		Bottom 20%		Top 20%		Bottom 20%	
Ranked indicator^1^	Min %	Max %	Min %	Max %	Min %	Max %	Min %	Max %
Mobility	0.8	4.7	15.3	31.5	0	4.5	13.3	28
Body condition	95.5	100	28.3	80.5	87.7	95.7	28.9	66.7
Ocular discharge	0	0	78.7	95.7	6.5	27.4	68.4	94
Nasal discharge	1.8	14.9	43.8	85.5	1.9	15.7	53.3	88.6
Tail lacerations	0	0	2.3	17.7	0	0	5.9	21.8
Tail breaks	0	2.8	13.6	51.6	0	1.8	14	47.3
Tail docks^2^	—	—	—	—	0	0	10	74.5
Integument	0	2.4	12.4	29.3	4.1	14.2	29.3	49.2
Avoidance								
Total	51	73.8	91.4	100	—	—	—	—
Pen	—	—	—	—	33.3	60	91.2	100
Feed-face	—	—	—	—	4	25	60.5	89.3

^1^Mobility, combined percentage of MS2 and MS3 scored cows; BCS, percentage of cows meeting the target levels of 2.75 to 3.25 at grazing and 3.0 to 3.5 for housing; ocular discharge, combined percentage of cows scoring OS1 to OS3; nasal discharge, combined percentage of cows scoring NS1 to NS3; tail lacerations, tail breaks, and tail docks, percentage of cows displaying each injury; integument, mean total percentage of integument alterations observed in all zones combined; avoidance, percentage of cows responding at levels 1 and 2 combined (Fearful response category).

^2^Docked tails were only recorded at VISIT2 when housed.

### Mobility

Lameness is a painful condition (Rushen et al., 2007) and widely considered one of the biggest risks to dairy cow welfare. During the grazing season (VISIT1), lame cows were present on all study farms with an average of 10% clinically lame (MS2 and MS3; Table 3). This is lower or comparable to the 14.6% and 11.6% lameness prevalence observed before and after the breeding season in a smaller study of Irish farms (Somers et al., 2015), or the 15% (Haskell et al., 2006) and 16% to 19% (Rutherford et al., 2009) reported for UK farms during grazing. However, using the same mobility scale, O′Connor et al. (2019) reported that 38% of cows on dairy farms in Ireland demonstrated signs of suboptimal mobility (MS1 to MS3) while grazing, which is considerably lower than the 64% of cows scored MS1 to MS3 in the current study. This is possibly due to variation in weather between study years, as wet conditions are shown to reduce claw hardness and potentially influence claw injuries (Borderas et al., 2004). Although “acceptable” levels of lameness vary within the literature, ideally, farms should aim for the lowest lameness prevalence possible within the herd, through early detection and treatment (Bell et al., 2009). Accordingly, the quality assurance scheme from the Irish Food Board, Bord Bia, recommends that farms “implement measures to minimize lameness” (Bord Bia Irish Food Board, 2013). The current study indicates that a low level of lameness is achievable during the grazing season within the Irish pasture-based system, with the top performing 20% of farms at VISIT1 displaying a mean of approximately 1% to 5% clinically lame cows (Table 2) including <1% severely lame cows. The bottom 20% of farms had between 15% and 32% clinically lame cows, indicating that there is considerable room for improvement among some farms.

**Table 3.** Descriptive statistics and means comparison of mobility scores for 82 spring-calving, pasture-based farms in southern Ireland during grazing (VISIT1) and when housed (VISIT2) in the 2019–2020 season^1^

	Mobility score^2^			
	0	1	2	3
Grazing				
Mean %	36	54	8.8	1.2
SEM	1.47	1.3	0.56	0.16
Min	9.8	22.7	0.8	0
Max	76.5	76.2	26.9	5.2
Median	34.9	54.6	8	0.7
Lower 95% CI	33.1	51.4	7.7	0.9
Upper 95% CI	39	56.6	9.9	1.5
Housed				
Mean %	39.6	51.1	8	1.3
SEM	1.36	1.25	0.52	0.19
Min	8	21.2	0	0
Max	78	74.8	23	8.1
Median	37.8	52	7.6	0.8
Lower 95% CI	36.9	48.7	6.9	0.9
Upper 95% CI	42.2	53.6	9	1.7
*P*-value^3^ (grazing vs. housed)	0.18^†^	0.30^†^	0.59^†^	0.95^‡^

^1^Mean of 123 cows/farm (range: 38 to 253 cows/farm) scored at VISIT1 (grazing) and 114 cows/farm (range: 40 to 232 cows/farm) scored at VISIT2 (housed).

^2^MS: 0) perfect mobility, 1) imperfect mobility, 2) impaired mobility/moderately lame, and 3) severely impaired mobility/severely lame.

^3^Bonferroni-corrected *P*-value for multiple comparisons of mean differences between VISIT1 and VISIT 2 (significant difference at *P* < 0.05). Tests were either paired *t*-tests for normally distributed variables (†) or Wilcoxon signed-rank tests for non-normally distributed variables (‡).

Cows in continuous-housing systems have demonstrated higher lameness prevalence than cows at pasture (Haskell et al., 2006). However, there was no significant difference observed in the mean percentage of lame cows of score MS2 or MS3 between grazing at VISIT1 (10%) and housing at VISIT2 (9%; Table 3). These values are considerably lower than lameness levels found in previous studies, of 17% for cows scored 3 wk into winter housing (Haskell et al., 2006) and 32.3% for cows scored a minimum of 2 wk into housing (de Vries et al., 2015). Levels in these studies are more similar to those of the lowest performing 20% of farms in the current study, which ranged from 13% to 28% clinically lame cows during housing. In contrast, the top performing farms at VISIT2 achieved levels of clinical lameness between 0% and 5% during housing (Table 2) with ≤1% severely lame cows. Both Haskell et al. (2006) and de Vries et al. (2015) suggest that time on pasture has a protective effect on mobility when transitioning from grazing to housing; thus, perhaps the lower relative proportion of lameness observed within this study is influenced by the long grazing season length of 240 d/yr on average.

Correcting for multiple comparisons, there were no observed differences in any MS level between visits (Table 3). However, a higher mean percentage of MS0 cows scored at VISIT2 compared with VISIT1 was detected before correction (*P* = 0.04), suggesting that a larger future study may reveal some differences in MS. Timing of visit had no effect on MS during the grazing period. Those farms visited in the second half of the housing period had a higher percentage of MS0 scored cows (period A mean = 34.0%, 95% CI: 30.8% to 37.2%; period B mean = 46.3%, 95% CI: 42.8% to 49.8%; *P* < 0.001) and a lower percentage of MS1 (period A mean = 56.5%, 95% CI: 53.7% to 59.4%; period B mean = 44.6%, 95% CI: 41.4% to 47.7%; *P* < 0.001) than those visited in the first half; there was no impact on MS2 or MS3. This suggests a reduction in cows with imperfect mobility over time housed. With fewer demands on cows during housing due to the cessation of lactation, such as shorter daily walking distances and lower pre-milking standing time, this may have resulted in reduced prevalence of mild claw disorders associated with imperfect mobility (O′Connor et al., 2019). This may also be influenced by a carryover effect from the cows’ time on pasture at the start of VISIT2, suggesting that future research is needed into the effects of the grazing period on imperfect mobility and mild claw disorders.

### Body condition

Variable quality and quantity of grass allowance at pasture may put cows at greater risk of developing metabolic issues such as negative energy balance, ketosis, and weight loss (Coleman et al., 2009; Mee and Boyle, 2020). Intakes of insufficient quantity or quality may also lead to a sensation of hunger, negatively impacting cows’ affective state (von Keyserlingk et al., 2009). Thus, maintaining appropriate body condition, monitored through regular scoring, is fundamental to ensuring good welfare in dairy cows. In the current study, the mean BCS across farms at VISIT1 was 3.1 ± 0.01 SEM (range: BCS 2.8 to 3.5). An average of 87% of cows were within the recommended target grazing BCS of 2.75 to 3.25 (Butler, 2016), with 2% of cows below target and 11% above (Table 4). Given that most of the cows whose score fell outside target BCS were over-conditioned, concerns that cows are not obtaining sufficient nutrition when grazing and are experiencing feelings of hunger generally appear to be unfounded. Although, this may still be a concern for those cows with low BCS (≤2.5), which constituted up to 14.9% of cows on farms during grazing and up to 10.6% of cows during housing (Table 4). The top 20% of farms were able to achieve 96% to 100% of cows within target levels at VISIT1 (Table 2).

**Table 4.** Descriptive statistics and means comparison of BCS for 82 spring-calving, pasture-based farms in southern Ireland during grazing (VISIT1) and when housed (VISIT2) in the 2019–2020 season^1^

	BCS^2^						
	≤2.5	2.75	3	3.25	3.5	3.75	4+
Grazing							
Mean %	2.2	16.1	49.5	21.8	7.2	2.8	0.5
SEM	0.31	1.27	1.57	1.38	0.68	0.57	0.18
Min	0	0	15	0	0	0	0
Max	14.9	50	84.2	50	22.9	43.3	11.7
Median	1.7	15	47.8	22	5.5	1.6	0
Lower 95% CI	1.6	13.6	46.3	19.1	5.8	1.6	0.2
Upper 95% CI	2.8	18.6	52.6	24.5	8.5	3.9	0.9
Housed							
Mean %	0.8	10.4	31.6	27.8	17.7	9.7	1.9
SEM	0.19	1.01	1.67	1.02	1.03	1.03	0.47
Min	0	0	2.6	5.3	0	0	0
Max	10.6	48	68	50	40	45.9	31.6
Median	0	8.4	29.8	26.6	17.5	6.7	0
Lower 95% CI	0.4	8.4	28.3	25.8	15.7	7.6	1
Upper 95% CI	1.2	12.5	34.9	29.9	19.8	11.8	2.8
*P*-value^3^ (grazing vs. housed)	<0.001^‡^	<0.01^†^	<0.001^†^	<0.01^†^	<0.001^†^	<0.001^‡^	<0.001^‡^

^1^Mean of 54 cows/farm (range: 33 to 76 cows/farm) scored at VISIT1 (grazing) and 52 cows/farm (range: 31 to 72 cows/farm) scored at VISIT2 (housed).

^2^Body condition scored on a 5-point scale with 0.25 increments from 1 (emaciated) to 5 (severely over-conditioned).

^3^Bonferroni-corrected *P*-value for multiple comparisons of mean differences between VISIT1 and VISIT 2 (significant difference at *P* < 0.05). Tests were either paired *t*-tests for normally distributed variables (†) or Wilcoxon signed-rank tests for non-normally distributed variables (‡).

Ensuring correct BCS at calving is key to ensuring cows remain healthy during the calving process and the following transition into lactation. Cows that are over-conditioned at calving are at greater risk of health problems, such as calving difficulties, excessive negative energy balance, milk fever, ketosis, left displaced abomasum, fatty liver, and retained fetal membranes (Atkinson, 2016). However, under-conditioning also leaves cows at greater risk of dystocia (Gearhart et al., 1990), lameness, retained fetal membranes, poorer fertility, and lower milk production (Atkinson, 2016). Therefore, it is critical to the health and welfare of the cow that her BCS during the indoor dry period is managed appropriately. At VISIT2, an average of 77% of cows on farms were within the recommended housing season targets (BCS 3.0 to 3.5; Butler, 2016) with a mean BCS of 3.2 ± 0.02 SEM (range: BCS 3.0 to 3.7). However, greater proportions within target levels are attainable; the top performing 20% of farms at VISIT2 achieved up to 96% of cows within target levels (Table 2). The percentage of thinner cows (mean percentage of cows with BCS ≤ 3) was lower while the percentage of heavier cows (mean percentage of cows with BCS ≥ 3.25) was greater at VISIT2 compared with VISIT1 (Table 4). Furthermore, for farms visited in the latter half of VISIT2, fewer cows with BCS 3.0 (period A mean = 36.4%, 95% CI: 32.2% to 40.6%; period B mean = 25.7%, 95% CI: 21.1% to 30.4%; *P* < 0.01) and more cows with BCS 3.5 (period A mean = 14.2%, 95% CI: 11.7% to 16.8%; period B mean = 22.0%, 95% CI: 19.1% to 24.8%; *P* < 0.001) were scored. This shift toward heavier cows is likely the result of scored cows having been dried-off for a longer period of time, thus providing cows more time to gain body condition. The higher proportion of cows outside target BCS levels (11% below and 12% above) at VISIT2 compared with VISIT1 may suggest greater difficulty in managing the feed requirements of cows transitioning from the lactating to the dry period, coinciding with the transition from grazed grass to grass silage as cows move indoors. It may also be influenced by greater competition for feed access when housed. Study farms had an average linear feed space of 0.52 m/cow (range: 0.21 to 1.19 m/cow), whereas the minimum recommended feed space allowance is 0.6 to 0.75 m/cow (Farm Animal Welfare Advisory Council, 2018). In competitive feeding environments, silage composition and the greater time required to consume a silage-based diet can negatively affect DMI (Grant and Ferraretto, 2018), potentially affecting some cows’ ability to maintain body condition. Competition for feed access can also promote feed sorting (Grant and Ferraretto, 2018), and as shown in studies of TMR diets, feed sorting can result in some cows, particularly subordinate individuals, consuming unbalanced diets when feeding at times further since fresh feed delivery (Leonardi and Armentano, 2003; Cook and Nordlund, 2009).

### Ocular and nasal discharge

There was no significant difference in the mean percentage of ocular discharge between grazing and housing visits, and most of the cows displayed either none or mild signs of discharge (Table 5). Additionally, the top 20% of farms achieved 0% of cows in the herd displaying any signs of ocular discharge at VISIT1 and between 7% and 27% of cows at VISIT2 (Table 2). Signs of moderate (OS2) and severe (OS3) ocular discharge were uncommon at either visit. The combined percentage of moderate and severe ocular discharge of approximately 1% on average in our study (Table 5) is well below both the “warning” and “alarm” thresholds of 3% and 6% proposed for equivalent score levels in Welfare Quality (2009). The “warning” threshold indicates that prevalence levels are approaching the “alarm” threshold, at which point implementing a herd health plan to manage the issue is needed. This suggests that health issues causing ocular discharge are not a major welfare concern on Irish farms.

**Table 5.** Descriptive statistics and means comparison of ocular and nasal discharge scores for 82 spring-calving, pasture-based farms in southern Ireland during grazing (VISIT1) and when housed (VISIT2) in the 2019–2020 season^1^

	Discharge score^2^			
	0	1	2	3
Ocular discharge				
Grazing				
Mean %	53.6	45	1.4	0
SEM	3.61	3.51	0.25	0.03
Min	4.4	0	0	0
Max	100	93.5	14.1	2
Median	46.8	51.7	0	0
Lower 95% CI	46.4	38	0.9	0
Upper 95% CI	60.8	52	1.9	0.1
Housed				
Mean %	53.5	45.2	1.2	0.1
SEM	2.31	2.21	0.27	0.08
Min	6	6.5	0	0
Max	93.6	92	11.4	4.9
Median	54.9	43.8	0	0
Lower 95% CI	48.9	40.8	0.6	0
Upper 95% CI	58.1	49.6	1.7	0.3
*P*-value^3^ (grazing vs. housed)	1.0^‡^	1.0^‡^	1.0^‡^	1.0^‡^
Nasal discharge				
Grazing				
Mean %	70	23.1	6.2	0.7
SEM	1.86	1.53	0.62	0.18
Min	14.5	0	0	0
Max	98.3	74.2	26.7	10.3
Median	72.3	21.2	4.4	0
Lower 95% CI	66.3	20.1	5	0.3
Upper 95% CI	73.7	26.2	7.5	1
Housed				
Mean %	64.7	30.6	4.4	0.4
SEM	2.23	1.85	0.65	0.12
Min	11.4	1.9	0	0
Max	98.2	67.1	31	6.1
Median	64.4	31	2.8	0
Lower 95% CI	60.2	26.9	3.1	0.1
Upper 95% CI	69.1	34.3	5.7	0.6
*P*-value^3^ (grazing vs. housed)	0.33^†^	0.02^†^	0.03^‡^	0.70^‡^

^1^Mean of 54 cows/farm (range: 33 to 76 cows/farm) scored at VISIT1 and 52 cows/farm (range: 31 to 72 cows/farm) scored at VISIT2.

^2^Ocular discharge score: 0) normal, 1) small amount of discharge, 2) moderate discharge, and 3) heavy discharge; nasal discharge score: 0) normal serous discharge, 1) small amount of cloudy discharge, 2) bilateral, cloudy, or excessive mucous discharge, and 3) copious bilateral mucopurulent discharge.

^3^Bonferroni-corrected *P*-value for multiple comparisons of mean differences between VISIT1 and VISIT 2 (significant difference at *P* < 0.05). Tests were either paired *t*-tests for normally distributed variables (†) or Wilcoxon signed-rank tests for non-normally distributed variables (‡).

Nasal discharge was absent in the majority of cows scored at either visit (Table 5). For cows that displayed nasal discharge, mild signs (NS1) were most prevalent and were present on 99% and 100% of farms at VISIT1 and VISIT2, respectively. The mean percentage of cows with mild nasal discharge was greater at VISIT2 than VISIT1, and the mean percentage with moderate discharge (NS2) was lower. The proportion of cows with moderate and severe (NS3) nasal discharge combined was approximately 7% on average at VISIT1 and 5% at VISIT2. This exceeds the Welfare Quality (2009) “warning” threshold of 5% for nasal discharge at VISIT1 and is equivalent to this threshold for VISIT2. Most of the previous welfare assessments of dairy cattle have reported no signs of nasal discharge (Zuliani et al., 2018) or no difference in the levels of nasal discharge between housing and pasture (Wagner et al., 2018). This suggests that there is some degree of compromised health of cows during both the grazing and housing periods. Potential causes of discharge include exposure to airborne contaminants, such as dust, gases, odors, and microorganisms (Casey et al., 2006); insects, such as flies (Reiten et al., 2018); or contagious disease, such as infectious bovine rhinotracheitis (Banks, 1999), for which only 60% of study farms vaccinated. However, levels of any signs of nasal discharge in 2% to 15% (during grazing) or 16% (during housing) of cows are achievable in this pasture system, as demonstrated by the top 20% of farms (Table 2).

### Tail injuries

Tail lacerations are characterized by deep, circumferential lacerations along the tail. With 33% of herds affected at VISIT1 and 43% at VISIT2, lacerations to the tail were somewhat common; however, affected cows were not widespread within those farms (Table 6), and the mean percentage of cows scored with lacerations was only 1% greater during VISIT2 than VISIT1. This lack of observed difference between visits suggests that there may be multiple causes of such injuries unrelated to the management period. Because 95% of study farms used automatic alley scrapers, to which cows were only exposed during housing, this potentially explains the observed numerical increase in tail lacerations. To prevent tail injury, some manufacturers recommend having a 1-inch buffer between the scraper’s edge and the curb, using a smoother edge to reduce entanglement in tail hair and ensuring that scrapers are properly maintained (GEA, 2019). The use of tail tape for marking cows (e.g., to indicate cows for breeding, dry-off, or grouping), practiced on 62% of the study farms, is another possible explanation for tail lacerations. Similar to what occurs during tail docking, where the application of a rubber ring prevents blood circulation to the distal portion of the tail (Sutherland and Tucker, 2011), the marking tape, if secured too tightly or left in place too long, could cause tissue damage that may result in the circumferential lacerations seen on some cows (DairyNZ, 2020). In this case, prevalence may be reduced by paying careful attention when applying tape to the tail so that circulation is not affected or by using alternative methods of identification.

**Table 6.** Descriptive statistics and means comparison of tail injuries for 82 spring-calving, pasture-based farms in southern Ireland during grazing (VISIT1) and when housed (VISIT2) in the 2019–2020 season^1^

	Tail injury				
	Lacerations	Breaks	Docks—all	Docks—short	Docks—long
Grazing					
Mean %	1.7	9.1	—	—	—
SEM	0.38	1.07	—	—	—
Min	0	0	—	—	—
Max	17.7	51.6	—	—	—
Median	0	7	—	—	—
Lower 95% CI	0.9	7	—	—	—
Upper 95% CI	2.4	11.2	—	—	—
Housed					
Mean %	2.8	8.5	7.5	2.6	4.9
SEM	0.56	1.03	1.53	0.66	1.25
Min	0	0	0	0	0
Max	21.8	47.3	74.5	40	72.6
Median	0	4.9	1.8	0	0
Lower 95% CI	1.7	6.5	4.5	1.3	2.4
Upper 95% CI	3.9	10.6	10.6	3.9	7.4
*P*-value^2^ (grazing vs. housed)	0.11^‡^	0.31^‡^	—	—	—

^1^Mean of 54 cows/farm (range: 33 to 76 cows/farm) scored at VISIT1 and 52 cows/farm (range: 31 to 72 cows/farm) scored at VISIT2.

^2^*P*-value for comparison of mean differences between VISIT1 and VISIT 2 (significant difference at *P* < 0.05). Tests were either paired *t*-tests for normally distributed variables (†) or Wilcoxon signed-rank tests for non-normally distributed variables (‡).

Tail breakage is a rarely documented injury in existing welfare assessment protocols (Laven and Jermy, 2020). Broken tails may result from mechanical damage (e.g., being stepped on or caught in manure scrapers) or from poor handling techniques, usually involving forceful tail twisting to motivate forward movement (Zurbrigg et al., 2005; Laven and Jermy, 2020). Understandably, there was no difference in the prevalence of tail breakage between visits as it constitutes a permanent injury (Table 6). However, at both visits, cows with tail breaks were present on 90% of farms, considerably higher than the 38% of the farms reported by Zurbrigg et al. (2005). Approximately, 9% of cows with broken tails were observed at both visits, which is similar to the annual herd prevalence of approximately 10% reported for pasture-based farms in New Zealand between 2014 and 2018 (Bryan et al., 2019). Research by Laven and Jermy (2020) determined that considerable force (9.8 to 20 Nm) was required to cause full vertebral dislocation and concluded that it was unlikely that such force could be applied accidentally while following recommended best practice for animal handling. It is possible that some injuries occurred as calves, when the force required to cause tail breakage is likely lower than that for an adult cow; however, this has not yet been studied. Further research is required to determine the cause and timing of such injuries in order to formulate preventative steps.

Tail docking is prohibited in Ireland according to Statutory Instrument No. 225/2014. Despite this, cows with either short or long docked tails were present on almost 65% of farms visited in this study, and 47% of cows with docked tails had entered the herd after this restriction was put into place. At VISIT1, we did not distinguish between short and long tail docking, so only the results from VISIT2 are reported (Table 6). Considering only those farms where cows with docked tails were observed (*n* = 53), long docking was present on 75% of farms and short docking on 62%. Research does not support claims that tail docking improves cow hygiene or prevents the spread of diseases such as mastitis (Tucker et al., 2001). Additionally, the removal of a cow’s tail impedes her ability to deter flies from her hind end (Sutherland and Tucker, 2011). The practice of tail docking is only acceptable for individual cows when medically necessary and performed by a veterinarian with the use of anesthesia. Such procedures may account for a low prevalence of docked tails on farms. However, we found that 33% of study farms had over 5% of the herd with docked tails, and 11% of farms had docked tails observed in 20% or more of the herd, suggesting that cows’ tails are being docked illegally for nonmedical reasons.

The performance of the top 20% of farms indicates that 0% of the herd with tail injuries due to lacerations, breaks, and docking is an achievable target (Table 2). However, there is a need for further study of the causes of tail injury in Irish dairy herds, methods of prevention, and better regulation of nonmedical tail docking to improve cow welfare in this area.

### Integument alterations

During the grazing period, only zone 2, corresponding with a cow’s hindquarters, displayed considerable integument alterations, both single and multiple to a similar degree (Table 7). Integument alterations to this zone were primarily mild, resulting in only hair loss (72 ± 3.3% SEM single mild alterations, range: 0% to 100%; 63 ± 4.2% SEM multiple mild alterations, range: 0% to 100%). This is likely due to frequent mounting behavior among cows in heat, as VISIT1 coincided with the breeding season. The common use of tail paint as a heat detection aid could contribute to hair loss on the hindquarters. The paint is designed to rub off as an indicator of mounting behavior (Diskin and Sreenan, 2000), and due to the paint’s thick consistency, it has been observed to simultaneously remove hair. On farms visited in the latter half of the VISIT1 period compared with the first half, fewer single alterations were scored for the head–neck–back (zone 1; period A mean = 6.9%, 95% CI: 4.3% to 9.5%; period B mean = 1.4%, 95% CI: −0.9% to 3.6%; *P* < 0.01), the hindquarters (zone 2; period A mean = 17.5%, 95% CI: 13.9% to 21.0%; period B mean = 8.8%, 95% CI: 5.7% to 11.9%; *P* < 0.01), the rear hocks (zone 3; period A mean = 6.2%, 95% CI: 4.3% to 8.0%; period B mean = 2.7%, 95% CI: 1.1% to 4.3%; *P* = 0.02), and the front hocks (zone 5; period A mean = 2.8%, 95% CI: 1.7% to 3.8%; period B mean = 0.6%, 95% CI: −0.3% to 1.5%; *P* < 0.01). Fewer multiple alterations were also scored for the hindquarters (zone 2; period A mean = 24.3%, 95% CI: 20.2% to 28.4%; period B mean = 4.6%, 95% CI: 1.0% to 8.2%; *P* < 0.001) on farms in the latter half of the VISIT1 period.

**Table 7.** Descriptive statistics and means comparison of integument alteration scores for all spring-calving, pasture-based study farms in southern Ireland during grazing (VISIT1) and when housed (VISIT2) in the 2019–2020 season^1^

	Zone 1^2^			Zone 2			Zone 3			Zone 4			Zone 5		
	N	S	M	N	S	M	N	S	M	N	S	M	N	S	M
Grazing															
Mean %	95.1	3.8	1.1	74.4	12.5	13.1	93.7	4.2	2.1	98.6	1	0.4	97.8	1.5	0.7
SEM	1.16	0.91	0.39	2.36	1.26	1.73	1.21	0.63	0.84	0.3	0.21	0.21	0.59	0.36	0.28
Min	43.6	0	0	14.3	0	0	35.4	0	0	84	0	0	66	0	0
Max	100	52.7	28	100	39.4	74.6	100	30.3	58.3	100	11.7	16	100	18.2	19.2
Median	98.3	1.5	0	79.2	7.8	6.3	98.1	1.8	0	100	0	0	100	0	0
Lower 95% CI	92.8	2.0	0.3	69.7	10	9.7	91.3	2.9	0.5	98	0.6	0	96.6	0.8	0.1
Upper 95% CI	97.4	5.6	1.9	79.1	15	16.6	96.1	5.5	3.8	99.2	1.4	0.9	99	2.2	1.3
Housed															
Mean %	34.2	45.9	19.9	67.8	13.7	18.5	90.4	8.3	1.3	96.8	2.5	0.6	94.7	3.8	1.5
SEM	2.78	2.65	2.71	2	0.74	1.73	1.51	1.26	0.35	0.5	0.4	0.15	0.82	0.47	0.57
Min	0	5.1	0	20.5	0	0	35.7	0	0	74.5	0	0	51.4	0	0
Max	92.5	92	92.9	98.1	32.2	64.1	100	58.9	20.5	100	19.6	5.9	100	17	41.4
Median	28.7	46.1	8.3	69.4	13.3	13.3	95.1	4	0	98.1	1.5	0	97.1	2.2	0
Lower 95% CI	28.7	40.6	14.5	63.8	12.2	15.1	87.4	5.8	0.6	95.8	1.8	0.3	93.1	2.9	0.4
Upper 95% CI	39.7	51.2	25.3	71.8	15.1	22	93.4	10.8	2	97.8	3.3	0.9	96.3	4.7	2.6
*P*-value^3^ (grazing vs. housed)	<0.001^‡^	<0.001^†^	<0.001^‡^	0.13^†^	1.0^†^	0.11^†^	0.22^‡^	0.05^‡^	1.0^‡^	<0.01^‡^	<0.001^‡^	0.26^‡^	<0.001^‡^	<0.001^‡^	0.40^‡^

^1^Mean of 54 cows/farm (range: 33 to 76 cows/farm) scored at VISIT1 (grazing) and 52 cows/farm (range: 31 to 72 cows/farm) scored at VISIT2 (housed). Integument was scored on a total of 81 farms at VISIT1 and 82 farms at VISIT2.

^2^One side of each scored cow was visually divided into five zones: 1) head–neck–back; 2) hindquarters; 3) lower rear legs; 4) flank, side, and udder; and 5) lower front legs. Each zone was scored for none (N), single (S), or multiple (M) areas of integument alteration.

^3^Bonferroni-corrected *P*-value for multiple comparisons of mean differences between VISIT1 and VISIT 2 (significant difference at *P* < 0.05). Tests were either paired *t*-tests for normally distributed variables (†) or Wilcoxon signed-rank tests for non-normally distributed variables (‡).

During housing, the majority of scored cows had no integument alterations in zones 2 to 5 (Table 7). The greatest percentage of all types of alterations were scored along the head–neck–back region (zone 1), followed by the hindquarters (zone 2), with the fewest alterations scored on the sides of the body (zone 4). Single alterations to the head–neck–back were the most common and primarily mild (98 ± 0.7% SEM, range: 61 to 100%) as were the multiple alterations (89 ± 3.1% SEM, range: 0% to 100%). When cows are housed indoors during the winter period, there is more opportunity for contact between the cows and housing elements, such as cubicles and feed rails, and thus have a greater risk of injury. Integument alterations to the neck are shown to be associated with feed barrier design (Kielland et al., 2010; Zaffino Heyerhoff et al., 2014). Ideal feed-rail height is related to cow size, with recommendations ranging from 1.1 m for smaller breeds, such as the Norwegian Red (Kielland et al., 2010), to upward of 1.3 or 1.4 m for the larger Holstein or Holstein-Friesian breeds (Huxley and Whay, 2006; Zaffino Heyerhoff et al., 2014). The average feed-rail height among current study farms ranged between 0.96 and 1.53 m, indicating that feed rails on some farms may fall outside recommended heights. Competition for feed access and infrequent feed delivery may be other contributing factors as they have also been associated with an increased risk of neck lesions (Kielland et al., 2010).

Generally, integument alterations were more prevalent during housing than while grazing (Table 7). The percentage of single alterations was higher at VISIT2 than VISIT1 in all zones except zone 2, the hindquarters, where no difference was observed. The percentage of multiple alterations was greater in only zone 1, the head–neck–back, at VISIT2 than VISIT1. However, prior to correction for multiple comparisons, a greater prevalence of multiple alterations was also detected for zone 2 (*P* = 0.04), potentially suggesting that a larger sample size in a future study may reveal more differences in integument condition. Similar levels of both single and multiple integument alteration to the hindquarters during grazing and housing indicate that multiple factors are responsible. In a spring-calving system, alterations to the hindquarters observed during housing cannot be attributed predominantly to estrus behavior as discussed for VISIT1. Rather, a cow’s hindquarters are likely more prone to contact with their surroundings while housed, since lesions in dairy cattle most commonly occur on protruding areas of the body such as the hips and pin bones (Weary and Taszkun, 2000). Considering all types of integument alterations combined across all zones of the body, the top 20% of farms were able to achieve between 0% and 2% alterations during grazing and 4% and 14% during housing (Table 2), reflecting the increased likelihood of integument damage experienced during housing.

The opportunity for injury due to housing features is greater with longer time spent in housing. Rutherford at al. (2008), for example, found an increase in hock damage of 34% between autumn and spring assessment. With the exception of the head–neck–back (zone 1), the observed differences were small, yet there was evidence of deteriorating integument condition during housing. At farms visited during the second half of VISIT2 compared with the first, more single alterations were observed on the rear hocks (zone 3, period A mean = 3.4%, 95% CI: 0.4% to 6.4%; period B mean = 14.3%, 95% CI: 11.0% to 17.6%; *P* < 0.001), the side body (zone 4, period A mean = 1.6%, 95% CI: 0.6% to 2.6%; period B mean = 3.7%, 95% CI: 2.5% to 4.8%; *P* = 0.03), and the front hocks (zone 5, period A mean = 2.5%, 95% CI: 1.3% to 3.7%; period B mean = 5.3%, 95% CI: 4.0% to 6.7%; *P* < 0.01). Higher percentages of multiple alterations were also scored for the head–neck–back (zone 1, period A mean = 12.9%, 95% CI: 5.9% to 19.8%; period B mean = 28.4%, 95% CI: 20.7% to 36.0%; *P* = 0.01), the hindquarters (zone 2, period A mean = 10.1%, 95% CI: 6.4% to 13.9%; period B mean = 28.8%, 95% CI: 24.7% to 32.9%; *P* < 0.001), and the rear hocks (zone 3, period A mean = 0.3%, 95% CI: −0.6% to 1.2%; period B mean = 2.5%, 95% CI: 1.5% to 3.5%; *P* < 0.01) at visits during the second half of VISIT2 than the first.

### Avoidance behavior

Avoidance distance of cattle from an approaching human is a measure of the human–animal relationship (Rousing and Waiblinger, 2004) and greater avoidance distance is indicative of more strain on this relationship. The cows’ responses to human approach (Table 8) were interpreted as Fearful (levels 1 and 2, retreat > 1 m from the observer), Intermediate (level 3, retreat < 1 m from the observer without extended hand), or Non-fearful (levels 4 and 5, accepting of hand or touch). A mean of 82% of cows on farms at VISIT1 were categorized Fearful, 13% Non-fearful, and 5% Intermediate. At VISIT2, those cows tested within the pen areas responded as 75% Fearful, 20% Non-fearful, and 5% Intermediate. Of the cows scored at the FF from outside the pen, 42% were categorized as Fearful, 47% Non-fearful, and 11% Intermediate. Fear response in dairy cattle is a natural aspect of their history as a prey species and serves to assist in avoiding potentially harmful situations (Rushen et al., 1999). However, disproportionate fear of humans can induce stress, negatively influencing a cow’s affective state, and may be an indicator of aversive handling (Pajor et al., 2000). A good human–animal relationship is crucial for maintaining the welfare of dairy cattle as contact with humans is an inevitable part of dairy management procedures and a fearful relationship can make handling more difficult and potentially dangerous for the cows and stockpersons (Rushen et al., 1999).

**Table 8.** Descriptive statistics and means comparison of avoidance behavior responses for spring-calving, pasture-based study farms in southern Ireland at the paddock during grazing (VISIT1) and inside the pen and at the feed-face when housed (VISIT2)^1^

	Response level^2^				
	1	2	3	4	5
Grazing					
Mean %	46	36	4.9	5.3	7.8
SEM	2.25	1.7	0.48	0.58	0.83
Min	14.3	12.2	0	0	0
Max	85.4	66.7	18.6	20	31.4
Median	45.6	36.8	4.1	4.1	5.9
Lower 95% CI	41.5	32.6	3.9	4.1	6.1
Upper 95% CI	50.5	39.4	5.8	6.4	9.5
Housed					
Pen					
Mean %	27.6	47	5.5	6.6	13.8
SEM	2.12	1.92	0.75	0.87	1.4
Min	0	15.4	0	0	0
Max	80.8	100	25	40	52.6
Median	23.6	45	3.9	5.6	12.1
Lower 95% CI	23.4	43.2	4	4.8	11
Upper 95% CI	31.8	50.8	7	8.3	16.6
Feed-face					
Mean %	8.1	33.5	11.1	19.8	27.5
SEM	1.1	1.85	1.03	1.38	1.67
Min	0	0	0	0	0
Max	35.7	80	36	50	65.2
Median	5	32.1	9.5	18.8	28.1
Lower 95% CI	5.9	29.8	9.1	17.1	24.2
Upper 95% CI	10.2	37.2	13.2	22.6	30.8
*P*-value^3^ (grazing vs. housed)					
Pen	< 0.001^†^	< 0.001^‡^	1.0^‡^	1.0^†^	< 0.001^‡^
Feed-face	< 0.001^†^	1.0^†^	< 0.001^†^	< 0.001^†^	< 0.001^†^

^1^Mean of 43 cows/farm (range: 30 to 54 cows/farm) scored at VISIT1 (grazing) and 44 cows/farm (range: 30 to 79 cows/farm) at VISIT2 (housed). Avoidance behavior data were available for a total of 68 farms at VISIT1 and 75 farms at VISIT2.

^2^The five levels of avoidance behavior indicate distance of individual cows’ retreat at: 1) >2 m from the observer, 2) within 1 to 2 m from the observer, 3) within 1 m from the observer but before extending hand, 4) accepting of hand but not touch, and 5) accepting of touch.

^3^Bonferroni-corrected *P*-value for multiple comparisons of mean differences between VISIT1 and VISIT 2 (significant difference at *P* < 0.05). Tests were either paired *t*-tests for normally distributed variables (†) or Wilcoxon signed-rank tests for non-normally distributed variables (‡).

Cows most commonly retreated from the observer at level 1 (>2 m) when approached in the paddock during grazing and at level 2 (within 1 to 2 m) when indoors during housing, both of which were categorized as Fearful responses. However, the proportion of animals exhibiting Fearful responses was generally lower during housing than when grazing, potentially because reduced space prevented them from retreating, or potentially because of more frequent close contact with farm staff. Greater familiarity with a person or object is known to result in a reduced flight zone in cattle (Grandin, 2017), and, during housing, cows are continually exposed to farm staff performing routine management, such as cleaning cubicles or delivering feed, resulting in greater familiarity over time. A positive human–animal relationship in cattle is built through continued experience of frequent, good quality interactions with stockpersons and reduction of aversive handling (Rushen et al., 1999; Rousing and Waiblinger, 2004). The top 20% of farms achieved Fearful category responses (levels 1 and 2 combined) in a maximum of 51% to 74% of the herd while grazing, which may serve as reasonable targets in pasture settings (Table 2). When cows were indoors, where there would be greater exposure to humans and cows were more likely to anticipate human interaction, the Fearful category response among the top 20% of study farms was lower at 33% to 60% when measured within the pen. This was further reduced to between 4% and 25% when measured at the FF. According to these benchmarks, we suggest that it should be considered unacceptable for any farms operating in this system to have more than approximately three-quarters of their herd retreat from humans at >1 m when grazing. Furthermore, when housed, it should be unacceptable to have more than two-thirds of the herd retreating at >1 m from humans within the pen or more than one-quarter of the herd at the FF.

There was a marked difference in the individual response levels when housed cows were scored from within the pen compared with when they were scored from the FF outside the pen. Overall, there was a lower percentage of level 1 responses at both the pen and the FF at VISIT2 than the paddock at VISIT1. Cows scored within the pen at VISIT2 displayed a higher percentage of level 2 and level 5 responses, and cows scored at the FF displayed a higher percentage of levels 3, 4, and 5 responses compared with VISIT1. Windschnurer et al. (2008) similarly found that cows were more often accepting of touch at the FF (41%) compared with within the pen (33%). It is possible that when feeding at the FF, the cows’ natural fear response as a prey species (Rushen et al., 1999) is reduced by the presence of the protective feed barrier between the cow and the observer, who may be seen as a threat. Additionally, research by Waiblinger et al. (2003) found that, when tested at the feed rail, avoidance behavior was most correlated to the level of agonistic social behavior. Thus, the lower fearful response to approach from outside the FF compared with within the pen could also be related to increased competition for feed access promoting greater agonistic interactions. In that case, the cows’ reluctance to forfeit their position at the FF may be overcoming their fearful response.

## Conclusions

The aim of this study was to provide a descriptive, exploratory analysis of welfare indicators for a dairy production system where little large-scale data are available. Throughout both the grazing and housing periods, Irish dairy farms in this study performed favorably in the area of lameness control compared with other studies, met recommendations for body condition management, and displayed signs of good ocular health. There is opportunity for improvement in dairy cow welfare through increased monitoring of housing facilities for potential sources of integument damage to the hindquarters. Areas were also identified that would benefit from further research. The cause of elevated levels of nasal discharge observed throughout the lactation is yet unclear. Signs of preventable or prohibited tail injury indicate a need to examine the causes, potential solutions, and enforcement of existing regulations. Furthermore, investigation into the impact of the level of competition for feed access on farms during housing is needed, as it may be linked to achieving target BCS levels, the prevalence of integument alterations on the head–neck–back region, as well as avoidance behavior. Finally, the identified targets for welfare indicators within Irish spring-calving, pasture-based dairy systems may benefit future research and may be used as benchmarks in the determination of future on-farm management and policy decisions for improving the overall welfare of dairy cows.

## Abbreviations

AHDB: Agriculture and Horticulture Development Board
BCS: body condition score
FF: feed-face
ICBF: Irish Cattle Breeding Federation
MS: mobility score
NS: nasal score
OS: ocular score
VISIT1: grazing period farm visit (April to September 2019)
VISIT2: housing period farm visit (October 2019 to February 2020)
